# Progress in General Surgery

**Published:** 1900-12-08

**Authors:** 


### Progress in General Surgery.

**Antiseptics.**—Regarding the value of alcohol as a disinfectant, particularly for the hands, Salzwedel and Elsner[1] report the result of experiments made by them in Koch's laboratory. The authors maintain that these researches show that alcohol is of use in preparing the hands for operations, not merely because of its hardening effect on the epidermis, but also as an active antiseptic; in this respect they assign it a potency intermediate between those of carbolic acid and corrosive sublimate. They recommend that the hands, after washing with soap and water, be well soaked in 80 per cent. acidulated spirit (which is diluted to the proper strength by the water remaining on the hands) before the performance of an operation. A new and simple method of sterilising catgut is described as follows by Dr. C. A. Elsberg.[2] The fatty matter is removed from the catgut by immersing it in ether or chloroform for from 12 to 48 hours. A mixture of chloroform one part, ether two parts, is advisable, as it seems to have greater penetrating power; besides chloroform is an antiseptic. The catgut when dry may be kept in stock if it is tightly wound in single layers on spools for sterilising. The spools are boiled for ten or twenty minutes in a hot saturated solution of ammonium sulphate in water, made by adding chemically pure ammonium sulphate to boiling water until no more will dissolve. When the spools are removed from the boiling solution some of the salt crystallises upon them. This is at once removed by a moment's immersion and agitation in warm or cold water, carbolic acid, or bichlorate solution. The catgut is then ready for use or for preservation in strong alcohol. Catgut thus prepared will be found to have lost none of its physical properties. It is as strong as catgut prepared by other methods, and experiments have shown that very fine catgut is stronger after having been boiled in the ammonium sulphate solution than after it has been prepared in other ways. The catgut does not swell up; it remains of the same thickness as the raw material. It is readily absorbed in the tissues of the body, and its absorption takes place between the fourth and eighth days. Bacteriological experiments have shown conclusively that catgut prepared by this method is always sterile after five minutes' boiling.

**Fractures and Dislocations.**—At the Hôtel Dieu, on August 6th, M. Lucas-Championnière[3] gave a lecture and demonstration (which have been referred to in The Hospital of August 25th) on the varieties of fractures in which the right treatment is massage and mobilisation, with suppression of fixed apparatus. He laid emphasis on the fact that a methodical, moderate movement is favourable to the setting of fractures and to the formation of firm callus. In addition to the statistics already quoted, Dr. A. S. Pendleton[4] notes that in ninety-seven cases so treated, in not one of them was an ununited fracture the resultant; that, from the results obtained in his own experience, he has no hesitation in declaring that there is no greater tendency to deformity than with any other treatment. He agrees in the belief that the immediate application of a plaster splint to a certain extent prevents swelling about the seat of injury, which when excessive retards union, and diminishes muscular spasm, which is so often a source of great pain after fracture. The early use of passive motion reduces to a minimum the stiffness resulting from treatment. As regards the ambulatory treatment of fractures, C. D. Lockwood[5] says that it was recommended by Berard as early as 1835. Among modern surgeons, Hessing was the first to successfully employ this method. The writer defines the ambulatory treatment as the application of such devices as will enable the patient to be put in an erect posture while bony union is taking place. He thinks that this method is especially applicable in fractures of the leg in children and those of the femur in adult persons, and in all simple and uncomplicated fractures of the lower extremity in young adults. The dangers attending ambulatory treatment are largely theoretical, and may be practically disregarded. The early application of ambulatory apparatus with massage is the ideal treatment for fracture. To an inquiry among London and provincial hospital surgeons, Mr. W. H. Bennett,[6] upon the methods at present available for the treatment of simple fractures the following facts were elicited. In fractures of the bones of the extremities, the large majority considered that no operation should be done except under special circumstances. Up to very recent date passive movement was never used until a very late stage. There was a general tendency to the disuse of plaster-of-Paris splints, as these tended to cause slow union and to give rise to prolonged disability. In fracture of the patella and of the olecranon, the answers to his special inquiry indicated that wiring the patella was by no means so routine a method of treatment as might be thought, and also that it was by no means free from danger to life and limb. Three surgeons out of 111 who gave information on the latter point had had fatal cases; eight had had to amputate; sixteen had had suppuration in the joints with ankylosis; five had had refracture occur or cutting through the bone by the wire during attempts at restoring movement, necessitating a second operation; and ten had had recurrent arthritis, necessitating removal of the wire. It was certain that the sooner passive movement was employed after wiring the sooner was the patient able to walk. As to the immediate use of massage, it appeared that most of the opposition to it was theoretical rather than practical. Some surgeons feared embolism, but embolism had occurred in cases of fracture where no massage had been employed. *The Lancet*,[7] in an article on the treatment of fractures, says that it is maintained that the lack of success which has been met with is attributable to a failure to adjust with accuracy the broken portions of the bones and to maintain them in position. Is the benefit to be derived from operation equal to the risk which is run? The affirmative answer can only be given in a small proportion of fractures. For an ordinary simple fracture the usual methods suffice, but the immediate result should be controlled by means of skiagraphy. Great care must be taken in replacing and fixing the bones, and after the splints have been applied the employment of the Röntgen rays will show their position, and will inform the surgeon what degree of success has been attained. Should the position be unsatisfactory, an attempt must again be made to replace them. If after repeated trials success should not be attained, more severe surgical measures are surely justified. G. Maunoury[8] says that the errors of radiography in the study of fractures and of dislocations were attributable to defective interpretation. To avoid such errors it was desirable to secure greater precision in the examinations, and to indicate on the prints the point where the vertical line let fall from the focus on to the plate meets the plate. In simple fractures radiography rendered invaluable service by indicating the number, form, and position of the fragments, their overriding and displacement in different directions, and the situation of splinters. The clinical diagnosis of fractures was as valuable as ever, but radiography complemented it very successfully by making it more definite. It made it less painful by reducing to a minimum the exploratory manœuvres at the seat of fracture. It is especially useful in fractures of the upper end of the humerus, of the lower end of the radius, of the leg, and especially those involving the tibio-tarsal articulation—in these last it can alone give exact information as to the relations of the astragalus with the tibio-fibular mortise, a vital point in the pronosis and treatment of these fractures—of the astragalus, which a few years ago were wrongly looked upon as very rare; lastly, of the metatarsal bones, which are the lesion of an affection now well known to military surgeons. Radiography also makes visible all the varieties of callus known to pathological anatomy, and shows how certain kinds of callus may simulate a perfect coaptation of two fragments which are, nevertheless, very badly reduced. It shows that in the child bony consolidation, instead of, as in the adult, being limited to the immediate neighbourhood of the fracture, spreads far along the line of fragments. By its means also the action of different splints and apparatus—immovable, for continuous extension, &c., can be studied. It indicates in what cases wiring of bones may be practised, especially in fractures with the joints, *e.g.*, the elbow and ankle; in such cases it enables us to see if the relations of the articular surfaces have been sufficiently re-established by reduction. After a short review of the literature of the thyroid treatment of non-union of fractures, Dr. F. W. Murray[9] adds a case of his own which was much benefited by this form of treatment. Steinlin has made some interesting experiments on animals to determine the influence of the thyroid body on the healing of fractures. As to the effect of the thyroid on the growth of bone, a most interesting question is seen in the beneficial action which thyroid medication exercises in cretinism and infantile myxœdema. Also, the introduction of the X-rays has made it possible to make a continued study of the progress of bony development in these affections, and to appreciate the action of thyroid medication in hastening ossification. Before deciding to use the thyroid the cause of the non-union should be considered in each case—whether it is due to some constitutional condition which may be corrected by appropriate medicine, or whether there may be some local cause which can only be removed by operation. Tabloids or capsules of two grains of the thyroid extract are sufficient to start with; then gradually increased. Disease of the heart and kidneys contra-indicates this form of treatment. With medical supervision, therefore, a trial of thyroid treatment may be recommended before proceeding to operative measures.

[1] Ep. Brit. Med. Jour., June 30, 1900, and Berl. klin. Woch., June 4, 1900. [2] New York Med. Rec., May 5, 1900, p. 760. [3] Lancet, Aug. 18, 1900, p. 448. [4] Charlotte Med. Jour., June, 1900, p. 577. [5] Clin. Jour., June 27, 1900, p. 160. [6] Lancet, Aug. 18, 1900, p. 498. [7] Lancet, May 26, 1900, p. 1532. [8] Ep. Brit. Med. Jour., Aug. 18, 1900, p. 27. [9] Annals of Surgery, June, 1900, p. 695.

